# Comparative study of the oral hygiene status of users of mefakia (traditional tooth cleaning method) and modern toothbrushes among patients at the Holy Bethel Dental Clinic in Addis Ababa, Ethiopia

**DOI:** 10.1038/s41405-024-00290-9

**Published:** 2024-12-28

**Authors:** Check Abebe, Yeshewas Abaynew

**Affiliations:** 1 Atlas College of Health Sciences, Addis Ababa, Ethiopia; 2 School of Public Health, College of Medicine and Health Sciences, Wollo University, Dessie, Ethiopia

**Keywords:** Dentistry, Oral diseases

## Abstract

**Background:**

Mefakia is a well-known traditional chewing wood used in Ethiopia to cleanse the mouth. Although mefakia is used in parallel with modern toothbrushes to improve oral hygiene, there is a gap in the literature regarding its comparative performance in removing plaque and maintaining good oral hygiene.

**Objective:**

This study aimed to evaluate and compare the oral hygiene status of patients using mefakia and modern toothbrushes at the Holy Bethel Dental Clinic in Addis Ababa, Ethiopia.

**Methods:**

This comparative cross-sectional study was conducted at the Holy Bethel Dental Clinic in Addis Ababa, Ethiopia. A total of 246 patients (123 mefakia and 123 modern toothbrush users) were included in this study. Participants were selected using a systematic random sampling method. Data on demographic characteristics, oral hygiene practices, and clinical oral health parameters, such as the calculus index, were collected through interviews and clinical examinations. Statistical analysis was performed using SPSS version 23 to compare the oral hygiene status between the two groups. The results are presented in tables, diagrams, and text.

**Results:**

Most respondents were aged 20–39; 66.7% and 73.2% used mefakia and toothbrushes, respectively. Sixty-seven percent of the toothbrush users had good oral hygiene, whereas 65% of the mefakia users had good oral hygiene.

**Conclusions:**

This finding suggests that mefakia and modern toothbrushes are comparable in their effectiveness in maintaining oral hygiene. Dental education should emphasize using available and affordable oral hygiene tools such as mechanical toothbrushes and fluoridated toothpaste to improve overall oral hygiene.

## Introduction

Oral health is the condition of the mouth, teeth, and orofacial structures that enable an individual to perform essential functions such as eating, breathing, and speaking. It also includes psychosocial dimensions, such as self-confidence, well-being, and the ability to socialize and work without pain, discomfort, and embarrassment [1].

Oral hygiene should be performed regularly to prevent dental diseases and bad breath. Viewing oral hygiene as a form of self-care not only emphasizes its importance but also empowers individuals to take responsibility for their dental health. The most common dental diseases are tooth decay and gum diseases such as gingivitis and periodontitis [2]. Dental diseases are among the most common noncommunicable diseases worldwide, affecting an estimated 3.5 billion people [1].

Poor oral health significantly impacts overall quality of life and well-being [3]. Several diseases, such as cardiovascular disease [4] and obesity [5], are associated with poor oral health. Maintaining oral hygiene by regularly removing plaque and food debris is considered a key factor in preventing poor oral health. With the correct technique, using the ‘ideal’ toothbrush, supplemented with fluoride toothpaste, at least twice a day for 2–3 min per session can effectively remove material alba and plaque from the mouth [6].

Mechanical cleaning methods are a reliable means of combating dental plaque, provided that cleaning is sufficiently thorough and carried out at regular intervals [7]. In many developing countries, natural methods of dental cleaning using chewing sticks are available, inexpensive, and simple [8]. Mefakia is an effective alternative to toothbrushing for maintaining oral hygiene, especially in countries where it is culturally significant and modern dental care is limited [9].

Mefakia not only is a means of oral hygiene but also has cultural and spiritual significance. It is believed to have healing properties and is used to treat various ailments, such as toothache, gum disease, and bad breath [10]. Mefakia is a traditional chewing stick widely used in Ethiopia, whose cultural significance goes beyond mere dental care and which embodies historical practices and social values in various Ethiopian communities [11].

Research has shown that, when used properly, mefakia can be as effective as toothbrushes in removing plaque and improving gum health. One study has shown that supervised use of mefakia in children resulted in significant improvements in oral hygiene, comparable to toothbrush use [12].

A randomized clinical trial conducted in Pakistan revealed that chewing sticks provide parallel and sometimes even stronger mechanical and chemical cleaning of oral tissues than toothbrushes do [13]. A 2003 study in Saudi Arabia comparing the use of miswak with conventional toothbrushes concluded that miswak is more effective than toothbrushes in reducing plaque and gingivitis when used properly [14].

A study conducted in Sweden concluded that miswak is as effective as a toothbrush in reducing plaque both experimentally and clinically [15]. A study conducted in Sudan examined the periodontal health of miswak users and suggested that their oral health status may be better than that of toothbrush users [16].

A cross-sectional study conducted in Ethiopia among patients attending the dental clinic of the University of Gondar Comprehensive Hospital revealed that 48.7% of the participants used a traditional stick called “Mefakia” for oral hygiene [17]. Another study in northwestern Ethiopia reported that in 29.9% of households, all family members regularly brushed their teeth with toothbrush sticks [11]. These findings highlight the prevalence of traditional oral hygiene practices in Ethiopia, indicating a significant reliance on mefakia among the population for cleaning their mouths.

In Ethiopia, few studies have compared the oral hygiene status of dental patients using traditional oral care (mefakia) and toothbrushes. This study aimed to fill this gap by conducting a comparative assessment of the oral hygiene status of Mefakia users and toothbrushes among dental patients at the Holy Betel Dental Clinic, thereby providing valuable insights into the effectiveness of traditional oral care methods in maintaining oral health.

## Materials and methods

### Study area and period

This study was conducted at the Holy Bethel Dental Clinic in Addis Ababa, Ethiopia. The clinic has four branches. It is a private dental care facility in an urban setting, catering to a predominantly diverse urban population of all ages, socioeconomic statuses, and educational levels. Some patients have access to some form of dental insurance. However, the clinic does not offer subsidized services to ensure affordability. The study was conducted from February 11, 2024, to March 13, 2024.

### Study design

This was an institutional, comparative, cross-sectional study.

### Population

The source population included dental patients attending the Holy Bethel Dental Clinic in Addis Ababa. In contrast, the study population included dental patients who visited the Holy Bethel Dental Clinic during the study period. Patients aged 18 years and above and mefakia or toothbrush users were included in the study. Patients with any systemic condition that may affect oral hygiene and those who used mefakia and toothbrushes were excluded from the study.

### Sample size determination and sampling methods

The sample size for the study was determined using Cochran’s Sample Size Formula for Comparing Two Proportions. The assumptions included a significance level (α) of 0.05, giving a critical value of 1.96, and a power (1 − β) of 0.80, giving a value of 0.84. An estimated population standard deviation of *σ*^2^ = 0.5 was used, along with an assumed minimum detectable difference (δ) of 0.5. The calculations result in n ≈ 123 participants for each group. Consequently, the calculated total sample size was 246, which ensures that the study has sufficient power to detect significant differences between the groups.

Study participants were selected using a systematic random sampling method. The sampling interval was determined by dividing the total number of eligible patients by the required sample size. The first participant was randomly selected from the first k patients. Patients were subsequently included for every 2nd patient after the first selection until the desired sample size was reached for each group (mefakia and toothbrush users). The study included a diverse sample to represent various socioeconomic backgrounds to provide a more comprehensive understanding of oral hygiene behaviors across different groups.

### Data collection

The data were collected using an interviewer-administered questionnaire and observations. A structured questionnaire was developed after a thorough literature review [9, 10, 12–14, 18]. The questionnaire included demographic data and oral hygiene practices (toothbrush or mefakia). A clinical examination was performed to assess the oral hygiene status of the participants using clinical parameters such as the plaque index with a dental probe and adequate lighting. Oral hygiene index-simplified (OHI-S) scores were recorded for each participant.

### Recording of oral hygiene status

Assessment of Oral Hygiene: The Simplified Oral Hygiene Index (OHI-S) was used to assess plaque and calculus in teeth #16 and #36 (Table 1).

**Table 1:** Summarizing the assessment of oral hygiene using the Simplified Oral Hygiene Index.

Tooth number	Surface assessed	Debris index (DI)	Calculus index (CI)	Interpretation
16 and 36	Facial or Buccal	0: No debris 1: Soft debris covering < 1/3 of the surface 2: Soft debris covering 1/3 to 2/3 of the surface 3: Soft debris covering > 2/3 of the surface	0: No calculus 1: Calculus covering < 1/3 of the surface 2: Calculus covering 1/3 to 2/3 of the surface 3: Calculus covering > 2/3 of the surface	OHI-S scores are interpreted as good (0–1.2), fair (1.3–3.0), or poor (3.1–6.0).

### Data entry and analysis

Data were entered, coded, and analyzed using the Statistical Package for the Social Sciences (SPSS) version 23 software. Descriptive statistics were used to summarize the data. The findings are displayed in texts and tables.

### Data quality assurance

The data collection tools were pretested before the main study for 5% of the sample size, and amendments were made based on the findings of the pretest. The data collectors were provided with training to improve the data collection. Data collection was supervised by a principal investigator.

### Ethical considerations

This study was approved by the Ethics Committee of the Atlas College of Health Sciences (ethics approval number: ACHS/039/24). A permission letter was submitted to the Holy Bethel Dental Clinic to obtain permission to conduct this study. Participants were given detailed information about the study’s objectives, procedures, potential risks, and benefits. Written informed consent was obtained from all the participants before the start of the study. The information was anonymized to protect the participants’ confidentiality.

## Results

### Sociodemographic characteristics of the participants

The majority of the respondents were in the 20–39 years age group, with 82 (66.7%) and 90 (73.2%) using mefakia and toothbrushes, respectively. Regarding sex, 72 (58.5%) males used Mefakia, while females used toothbrush. In terms of educational status, 52 (42.3) mefakia users and 66 (53.7%) toothbrush users had a college degree or higher. About 39.8% of mefakia users and 52.8% of toothbrush users had a monthly income of more than 5000 Ethiopian Birr (ETB) (Table 2).

**Table 2:** Sociodemographic characteristics of the participants at the Holy Bethel Dental Clinic, Addis Ababa, 2024.

Variables	Method for maintaining oral hygiene	
	Mefakia *n*(%)	Toothbrush *n*(%)
Sex		
Male	72 (58.5)	57 (46.3)
Female	51 (41.5)	66 (53.7)
Age group		
10–19	9 (7.3)	10 (8.1)
20–29	82 (66.7)	90 (73.2)
30–39	28 (27.2)	19 (15.4)
>39	4 (3.3)	4 (3.3)
Educational level		
Illiterate	15 (12.2)	6 (4.9)
Primary	22 (17.9)	16 (13)
Secondary	34 (27.6)	50 (40.7)
College degree and more	52 (42.3)	51 (41.5)
Occupation		
Unemployed	15 (12.2)	7 (5.7)
Student	20 (16.3)	15 (12.2)
Government employee	50 (40.7)	60 (48.8)
Private employee	38 (30.8)	41 (33.3)
Monthly income		
<1000 ETB	11 (8.9)	11 (8.9)
1000–2999 ETB	28 (22.8)	28 (22.8)
3000–4999ETB	35 (28.5)	19 (15.4)
>5000ETB	49 (39.8)	65 (52.8)

### Oral hygiene status of the participants

Of the study participants, 21 (17.1%) Mefakia users and eight (6.5%) toothbrush users regularly visited the Holy Bethel Dental Clinic for dental care. Of the study participants, 42 (34.1%) Mefakia users and 30 (24.4%) toothbrush users reported that the cost of dental care influenced the frequency of their dental visits (Table 3). Of the study participants, 81 (65.9%) Mefakia users and 82 (66.7%) toothbrush users had good oral hygiene (Table 4).

**Table 3:** Patient report on their perceptions of the effectiveness of various oral hygiene practices at the Holy Bethel Dental Clinic, Addis Ababa, 20249.

Variables	Method for maintaining oral hygiene	
	Traditional oral care (mefakia) *n* (%)	Toothbrush *n* (%)
Visit the Holy Bethel Dental Clinic for dental care.		
Rarely	72 (58.5)	83 (67.5)
Occasionally	30 (24.4)	32 (26)
Regularly	21 (17.1)	8 (6.5)
How would you rate the current health of your gums?		
Excellent	13 (10.6)	12 (9.8)
Good	27 (22)	27 (22)
Poor	38 (30.9)	32 (26)
Very poor	45 (36.6)	52 (42.3)
How effective is traditional oral care (Mefakia) compared to using a toothbrush		
Much less effective	49 (39.8)	35 (28.5)
Less effective	24 (19.5)	31 (25.2)
Equally effective	28 (22.8)	26 (21.1)
More effective	15 (12.2)	17 (13.8)
Much more effective	7 (5.7)	14 (11.4)
What factors, if any, influence your frequency of dental care visits?		
Cost	42 (34.1)	30 (24.4)
Time	34 (27.6)	37 (30.1)
Fear	28 (22.8)	37 (30.1)
Other	19 (15.4)	19 (15.4)

**Table 4:** Oral hygiene status of the participants at the Holy Bethel Dental Clinic, 2024.

Variables	Method for maintaining oral hygiene	
	Traditional oral care (mefakia) *n* (%)	Toothbrush *n*(%)
How often do you change your current method for maintaining oral hygiene?		
After 3 months	17 (13.8)	13 (10.6)
After 6 months	41 (33.3)	41 (33.3)
After 1 year	37 (30.1)	31 (25.2)
Other	28 (22.8)	38 (30.9)
How long have you been using your current oral hygiene method?		
Less than 6 months	14 (11.4)	22 (17.9)
6 months to 1 year	33 (26.8)	26 (21.1)
1 to 2 years	44 (35.8)	35 (28.5)
More than 2 years	27 (22)	37 (30.1)
Have you ever experienced any adverse effects?		
Yes	38 (30.9)	57 (46.3)
No	85 (69.1)	66 (53.7)
How comfortable do you feel with your current oral hygiene routine?		
Very uncomfortable	15 (12.2)	7 (5.7)
Uncomfortable	20 (16.3)	15 (12.2)
Neutral	44 (35.8)	40 (32.5)
Comfortable	25 (20.3)	35 (28.5)
Very comfortable	19 (15.4)	26 (21.1)
Would you be open to trying a different oral hygiene method if recommended		
Yes	76 (61.8)	55 (44.7)
No	47 (38.2)	68 (55.3)
Oral hygiene status		
Good oral hygiene	81 (65.9)	82 (66.7)
Fair oral hygiene	27 (22)	28 (22.8)
Poor oral hygiene	15 (12.2)	13 (10.6)

## Discussion

This study compared the effectiveness of mefakia and modern toothbrushes in patients attending the Holy Betel Dental Clinic in Addis Ababa. This study provides evidence of the effectiveness of traditional oral hygiene remedies in maintaining oral hygiene in culturally significant contexts in Ethiopia.

The results of this study revealed that 67% of the patients who used toothbrushes had good oral hygiene, whereas 65% of the participants who used mefakia had good oral hygiene. These results show that oral hygiene is equally effective for users of both methods. This finding aligns with the results of previous studies suggesting that mefakia can offer similar benefits to modern oral hygiene products. For example, a study conducted in Saudi Arabia concluded that the periodontal status of miswak users was similar to that of toothbrush users, suggesting that the efficacy of miswak use for oral hygiene is comparable to that of modern toothbrushes [19]. This finding is consistent with a study conducted in Asella, Ethiopia, which revealed that miswak is as effective as toothbrushes in removing oral debris, supporting its inclusion in dental prevention programs [12]. A cross-sectional study conducted in Pakistan revealed no significant difference in plaque index scores between miswak and toothbrush users, suggesting that both are equally effective at cleaning teeth when used correctly [18]. These results suggest that both toothbrushes and traditional oral care methods are equally effective in maintaining good oral hygiene. This study revealed that users of traditional oral care (mefakia) had almost identical oral hygiene statuses as did toothbrush users at the Holy Betel Dental Clinic.

However, a study comparing the effects of chew sticks and toothbrushes on plaque removal and gum health concluded that a miswak is more effective than toothbrushes in reducing plaque and gingivitis, especially when users receive professional instructions on proper use. The study revealed a significant reduction in plaque and gingivitis indices in miswak users compared with toothbrush users [14]. This discrepancy could be due to differences in the study populations included in the study and the measurements used in the studies.

The percentage of patients with good oral hygiene was similar between those who used a toothbrush (67%) and those who used traditional oral care methods, such as mefakia (65%). These findings suggest that traditional oral hygiene methods may be as effective as toothbrushes with fluoridated toothpaste for maintaining good oral health. The results also suggest that traditional oral care methods, such as mefakia, could be included in dental prevention programs and recommended alongside modern toothbrushes, as they appear to be equally effective in maintaining oral hygiene. This argues for the continued use and possible integration of traditional practices into dental care and prevention programs. However, it is important to note that while brushing is an essential part of oral hygiene, it should be complemented by other practices such as flossing, mouthwash, and regular dental check-ups for comprehensive oral health care. This study found that 21 participants (17.1%) who used mefakia and 8 participants (6.5%) who used modern toothbrushes reported regular dental check-ups. This result underlines the importance of regular dental visits in maintaining overall oral health.

This study had several limitations. First, the study did not adequately address the issue of sample representativeness. This study only included participants from a single clinic, which can affect the generalizability of the findings. Furthermore, this study was based on self-reporting and there were concerns about the accuracy and reliability of the information collected. There is a lack of comprehensive data collection to account for other factors that could affect oral hygiene, such as dietary habits, access to dental care, and cultural practices, which could undermine the validity of the conclusions. In addition, a cross-sectional design was used in this study, which precludes the possibility of establishing causal relationships between the variables.

## Conclusions

This study provides valuable insights into the effectiveness of traditional and modern oral hygiene practices in a culturally significant context in Ethiopia. The proportion of participants with good oral hygiene among modern toothbrush and mefakia users suggests that the two oral hygiene methods are comparably effective. This study emphasizes the importance of promoting effective oral hygiene methods, whether through traditional methods such as mefakia or fluoridated toothpaste. Encouraging the proper use of mefakia and modern dental care could improve the population’s oral hygiene. Dentists should consider the cultural context and patient preferences when recommending oral hygiene methods while guiding the effective use of traditional and modern tools for optimal oral hygiene. As a recommendation for future research, more detailed data should be collected, including dietary habits, access to dental care, and cultural practices, to understand how such factors may influence the use of mefakia and modern toothbrushing techniques. In addition, future research should be conducted using qualitative methods to explore oral health trends and dental health-seeking behaviors in different contexts in Ethiopia. This study aimed to assess oral hygiene. Future studies should consider differences in oral health measures, such as decayed teeth or periodontal status.

## Data Availability

Data supporting the results of this study are available upon reasonable request from the corresponding author.
